# Mechanistic insights revealed by lipid profiling in monogenic insulin resistance syndromes

**DOI:** 10.1186/s13073-015-0179-6

**Published:** 2015-06-28

**Authors:** Michael Eiden, Albert Koulman, Mensud Hatunic, James A. West, Steven Murfitt, Michael Osei, Claire Adams, Xinzhu Wang, Yajing Chu, Luke Marney, Lee D. Roberts, Stephen O’Rahilly, Robert K. Semple, David B. Savage, Julian L. Griffin

**Affiliations:** MRC Human Nutrition Research, Elsie Widdowson Laboratory, 120 Fulbourn Road, Cambridge, CB1 9NL UK; Metabolic Research Laboratories, Institute of Metabolic Science, University of Cambridge, Cambridge, UK; Department of Biochemistry and the Cambridge Systems Biology Centre, University of Cambridge, Tennis Court Road, Cambridge, UK

## Abstract

**Background:**

Evidence from several recent metabolomic studies suggests that increased concentrations of triacylglycerols with shorter (14–16 carbon atoms), saturated fatty acids are associated with insulin resistance and the risk of type 2 diabetes. Although causality cannot be inferred from association studies, patients in whom the primary cause of insulin resistance can be genetically defined offer unique opportunities to address this challenge.

**Methods:**

We compared metabolite profiles in patients with congenital lipodystrophy or loss-of-function insulin resistance (*INSR* gene) mutations with healthy controls.

**Results:**

The absence of significant differences in triacylglycerol species in the INSR group suggest that changes previously observed in epidemiological studies are not purely a consequence of insulin resistance. The presence of triacylglycerols with lower carbon numbers and high saturation in patients with lipodystrophy suggests that these metabolite changes may be associated with primary adipose tissue dysfunction. The observed pattern of triacylglycerol species is indicative of increased de novo lipogenesis in the liver. To test this we investigated the distribution of these triacylglycerols in lipoprotein fractions using size exclusion chromatography prior to mass spectrometry. This associated these triacylglycerols with very low-density lipoprotein particles, and hence release of triacylglycerols into the blood from the liver. To test further the hepatic origin of these triacylglycerols we induced de novo lipogenesis in the mouse, comparing ob/ob and wild-type mice on a chow or high fat diet, confirming that de novo lipogenesis induced an increase in relatively shorter, more saturated fatty acids.

**Conclusions:**

Overall, these studies highlight hepatic de novo lipogenesis in the pathogenesis of metabolic dyslipidaemia in states where energy intake exceeds the capacity of adipose tissue.

**Electronic supplementary material:**

The online version of this article (doi:10.1186/s13073-015-0179-6) contains supplementary material, which is available to authorized users.

## Background

Insulin resistance (IR) is a major factor in the development of type 2 diabetes mellitus (T2DM) and underpins the tight relationships among obesity and many of its metabolic complications, including hyperglycaemia, high triacylglycerol (TAG) levels, low high-density lipoprotein (HDL)-cholesterol, non-alcoholic fatty liver disease (NAFLD), and hypoadiponectinaemia as well as hyperandrogenism in women. Although progress has been made in understanding the molecular basis of human IR, considerable uncertainty remains. Obesity is the major association with the recent increase in IR incidence, so understanding the mechanistic basis of this association is a priority. Genetic susceptibility also plays an important role, but results to date from population-wide genetic studies, and the well-established heterogeneity seen even in the known Mendelian forms of T2DM, argue that determining the nature of this is likely to be very challenging.

Lipidomic profiling in epidemiological and cohort studies has recently suggested that both non-esterified fatty acids and fatty acids covalently linked to glycerol in TAGs tend to be shorter and more saturated, containing palmitate and stearate, in insulin resistant people, and that the TAG profile is another significant predictor of future T2DM [1, 2]. More recently, Stegemann and colleagues [3] have associated these TAG profiles with an increased relative risk of developing cardiovascular disease (CVD) in the prospective population-based Bruneck study (685 individuals analysed at baseline in 2010). These results were further confirmed in the UK Twin Registry [3]. More generally, T2DM, CVD and metabolic diseases have been associated with major remodelling of lipid profiles in plasma, suggesting that either lipid classes or specific lipid species could be used to monitor disease progression and treatment efficacy [4–6]. In purely epidemiological studies, however, the associations do not truly probe the mechanisms responsible for the association of a given lipid species with the risk of developing T2DM or CVD. Furthermore, such approaches require relatively large cohorts and place significant demands on the analytical approaches used to molecularly phenotype individuals. An alternative approach is to investigate the phenotype of rare genotypes where the underlying causes of IR can be discerned.

Mendelian randomization is increasingly used to address this problem of causality in associations, both in large epidemiological cohorts and in rarer monogenic disorders. Critical to this is the unequivocal direct link of a congenital genetic defect to a perturbation of the putative primary phenomenon of interest, in this case IR, without any direct effect on the associated trait being tested. If this condition holds, then demonstrating association between the IR-causing genetic variant and this second trait provides strong evidence that it is caused by IR. We and others have previously employed this strategy in efforts to understand the relationship between mitochondrial dysfunction and IR [7, 8]. Here, we report plasma lipidomic profiling results for a cohort of patients with severe IR due to either congenital lipodystrophy (LD), a state characterized by primary adipose tissue dysfunction, or loss-of-function mutations in the genes encoding the insulin receptor signalling pathway, sometimes referred to as ‘insulin receptoropathies’ (INSR). Thus, using these groups of rare inherited forms of IR, one can examine molecular changes associated with LD, and hence adipose tissue dysfunction, or molecular events associated with INSR to examine the consequences of a primary deficit in insulin signalling. Furthermore, molecular events that are common to both LD and INSR are presumably downstream of the genetic causes of both INSR and LD. In this manner one can begin to deduce what is upstream or downstream in terms of the causes of IR.

In the present study, the lipidomic profile of the INSR group was largely normal, whereas that of the LD group was associated with a relative increase in TAGs containing shorter (14–18 carbon atom fatty acids) and more saturated fatty acids. Furthermore, we show that these TAGs are exported by the liver in very-low-density lipoprotein (VLDL), and that similar changes are observed when de novo lipogenesis (DNL) is stimulated in the mouse by increasing consumption of a diet rich in carbohydrates. Finally, we suggest that the increase in these TAGs in previously reported epidemiology studies may be associated with increased DNL in the liver, aggravating NAFLD and IR, prior to either the development of T2DM or CVD.

## Methods

### Patient sample collection

Clinical studies were approved by the National Health Service Research Ethics Committee UK. Each participant provided written informed consent, and all studies were conducted in accordance with the principles of the Declaration of Helsinki.

Genetically characterised patients were identified as part of a longstanding programme of research into genetic and acquired forms of severe IR. Fasting blood samples were obtained from patients with severe IR caused by either loss-of-function mutations in the insulin signalling pathway or congenital generalised or partial LD. Clinical and biochemical features of these participants are summarized in Table 1. Healthy control volunteers were recruited by advertisement. All controls were non-smokers, without any medical conditions likely to influence energy balance and without a family history of diabetes. Participants were admitted to the Wellcome Trust Clinical Research Facility in Cambridge the night before sampling where they were provided with an energy balance meal of standard macronutrient composition at 6 p.m. They then fasted overnight prior to blood sampling the following morning.

**Table 1:** Characteristics of the healthy volunteers (controls) and patients with INSR mutations or LD

	Controls	Insulin receptoropathies	Lipodystrophy	*P* value
Gender	15 F, 12 M	6 F, 1 M	12 F, 2 M	
Age (years)	39 ± 14	27 ± 11	36 ± 9	0.14
BMI (kg/m^2^)	24.6 ± 2.6	23.2 ± 3.6	24.1 ± 2.4	0.50
Fat mass (kg)	20.9 ± 8.5	22.6 ± 10.0	10.2 ± 5.0	<0.01
Lean mass (kg)	47.3 ± 10.8	34.2 ± 3.8	53.9 ± 10.7	<0.01
Glucose (mmol/l)	4.5 ± 0.5	4.7 ± 0.8	6.4 ± 2.7	<0.01
Insulin (pmol/l)	27 ± 20	419 ± 269	116 ± 65	<0.01
Adiponectin (mg/l)	6.4 ± 3.2	10.5 ± 7.9	2.9 ± 3.3	<0.01
Leptin (μg/l)	16.9 ± 22.1	16.1 ± 8.1	4.2 ± 3.0	0.24
Triglyceride (mmol/l)	0.9 ± 0.4	1.0 ± 0.3	2.9 ± 2.3	<0.01
HDL-cholesterol (mmol/l)	1.5 ± 0.4	1.8 ± 0.4	0.9 ± 0.5	<0.01

Data presented are mean ± standard deviation. *BMI* body mass index, *F* female, *HDL* high-density lipoprotein, *M* male

### Mouse study of diet-induced DNL

Male ob/ob mice and their wild type (C57Bl/6J) were housed in a temperature- and humidity-controlled facility, with a 12 h:12 h light–dark cycle and access to water *ad libitum*. Mice (n = 10/age group/genotype) fed on a regular chow diet (caloric content of 11.5 % fat, 26.9 % protein, 61.6 % carbohydrate) were sacrificed at the age of 4 months. A separate group of mice was switched to a custom-produced high fat diet (caloric content of 55% fat, 29% protein, 16% carbohydrate) at 12 weeks and sacrificed at the age of 4 months. Blood plasma was collected by cardiac bleed and liver tissue collected and rapidly frozen post mortem. Blood plasma (15 μl) and liver tissue (100 mg) were stored at −80 °C until further analysis. All animal protocols were approved by the UK Home Office.

### Lipidomic analysis by Direct Infusion Mass Spectrometry (DIMS)

All solvents used were of liquid crystallography (LC)-mass spectrometry (MS) grade or better and ordered from Sigma Aldrich (Gillingham, UK). All internal standards were obtained from Avanti Polar Lipids with the exception of undecanoic acid and trilaurin (Sigma Aldrich, Gillingham UK).

Human and mouse plasma samples (15 μl) were extracted using an automated Anachem Flexus sample preparation unit (Anachem, Milton Keynes, UK) to minimize experimental variation. A total of 48 blood plasma samples were included for the human study, with the remaining positions of the 96-well plate occupied by either blanks or quality control (QC) samples. The samples, QC samples and blanks were placed in a pre-defined random order across the 96-well plate. For the QC samples, a pooled aliquot was created by mixing 10 μl of all samples to create the QC. Each plasma sample was placed into 1.2 ml Cryovials for the Flexus to sample. To each vial 100 μl of MilliQ water was added and thoroughly mixed. The solution (100 μl) was transferred to a glass coated 2.4 ml deep well plate (Plate+, Esslab, Hadleigh, UK). CH_3_OH (250 μl) was added containing six internal standards (0.6 μM 1,2-di-o-octadecyl-sn-glycero-3-phosphocoline, 1.2 μM 1,2-di-O-phytanyl-sn-glycero-3-phosphoethanolamine, 0.6 μM C8-ceramide, 0.6 μM N-heptadecanoyl-D-erythro-sphingosylphosporylcholine, 6.2 μM undecanoic acid, 0.6 μM trilaurin), and then 500 μl of methyl tert-butyl ether was added and mixed. The plates were sealed using Corning aluminium microplate sealing tape (Sigma Aldrich, UK) and shaken for 10 min at 600 rpm, after which the plate was transferred to a centrifuge and spun for 10 min at 6000 rpm. Each well in the resulting plate had two layers, with an aqueous layer at the bottom and a hydrophobic layer on top containing the lipid extract. A 96-head micro-dispenser (Hydra Matrix, Thermo Fisher Ltd, Hemel Hampstead, UK) was used to transfer 25 μl of the hydrophobic layer to a glass-coated 25 μl low well plate (Plate+, Esslab, Hadleigh, UK) and with 90 μl of 7.5 mM NH_4_Ac IPA:MeOH (2:1) using a Hydra Matrix, after which the plate was sealed using Corning aluminium microplate sealing tape (Sigma Aldrich, UK) and stored at −20 °C until analysis.

Lipidomics was performed on the extract using chip-based nanoelectrospray with an Advion TriVersa Nanomate (Advion, Ithaca, USA) interfaced to the Thermo Exactive Orbitrap (Thermo Scientific, Hemel Hampstead, UK), using a mass acquisition window from 200 to 2000 m/z and acquisition in positive and negative mode. The Nanomate infusion mandrel was used to pierce the seal of each well before analysis, after which a fresh tip was pre-wetted by three repeat mixes of 6.5 μl of the sample and 5 μl was aspirated, followed by an air gap (1.5 μl). The tip was pressed against a fresh nozzle and the sample was dispensed using 0.3 psi nitrogen pressure. A voltage of 1.2 kV was used in positive mode and −1.5 kV in negative mode with an acquisition time of 72 s in both modes and a total analysis time of ~3 min per sample. The tip was discarded and replaced for each sample to minimise sample carry-over. Throughout the acquisition the sample plate was kept at 15 °C.

Acquired spectral raw data were processed using an in-house bioinformatics platform based on XCMS [9]. This performed sample-specific mass re-calibration using predefined sets of internal standards and the removal of commonly present contaminant ions (often associated with plasticizers) with help of predefined rejection lists and mass defect filters. The raw data were converted to .mzXML (using MSconvert [10] with peakpick level 1), parsed with R and 50 (scan from 20 to 70) spectra were averaged per sample using XCMS [9], with a signal cutoff at 2000. The files were aligned using the XCMS [9, 11] grouping function using “mzClust” with a m/z-window of 22 ppm and a minimum coverage of 60 %, giving 1699 signals. Isotopes were annotated using the CAMERA package in R with the following parameters (maxcharge = 1, ppm = 5, mzabs = 0.001, intval = c(“into”), minfrac = 0.25) [11].

Automated compound annotation was carried out using both exact mass search in compound libraries as well as applying the referenced Kendrick mass defect approach. Features of interest were subsequently confirmed using fragmentation experiments on a Thermo Velos Orbitrap mass analyser using an Advion Nanomate to directly infuse the lipid extract. The selected masses were isolated with a 1.5 m/z width in the linear ion trap and then fragmented using either linear ion trap with 35% relative collision energy or in the HCD (higher-energy collision-induced dissociation) collision cell, with a range of collision energies from 5% to 75% relative collision energy. All spectra were recorded in the Orbitrap set at 100,000 resolution.

All human DIMS data have been deposited at the repository MetaboLights [12] (accession number MTBLS162).

### Size exclusion chromatography of human blood plasma

Freshly thawed plasma samples were diluted with phosphate-buffered saline (PBS; 1 volume plasma:5 volumes PBS). Lipoprotein particles were separated on a Shimadzu Prominence UFLC system using a Superose 6 PC 3.2/30 column (GE Healthcare Europe GmbH, Munich, Germany) with PBS as a mobile phase (at 50 μl min^−1^). The eluent was led to a Syrris FLLEX module (Syrris, Royston, Herts, UK), which also received CHCl_3_/MeOH (3:1) at 50 μl min^−1^. Using a 250 μl loop the two phases were mixed and then separated using a Teflon membrane in the pressurized glass flow chamber of the FLLEX module. This produced both the aqueous and organic phases as separated eluents. The organic phase was mixed with 1:1 isopropanol/methanol containing 7.5 mM ammonium acetate at 150 μl min^−1^ and led to a standard electrospray source on a LTQ Orbitrap Velos for the acquisition of mass spectra.

### RT-PCR of transcripts involved in de novo lipogenesis

Total RNA was extracted from 15 mg of mouse liver using the RNeasy lipid tissue kit (Qiagen, Hilden, Germany) following the manufacturer’s protocol, and the mRNA diluted in RNase free water. The amount of RNA extracted was measured on a NanoDrop spectrophotometer (Thermo Fisher Scientific, USA) and the purity of the mRNA determined by measuring the A_260_/A_280_ ratio on a spectrophotometer. mRNA was converted into cDNA using the Quantitect reverse transcription kit (Qiagen, Hilden, Germany) following the manufacturer’s guidelines. cDNA was diluted in RNase-free water to 50 ng of cDNA as a template for amplification. RT-PCR was carried out using the TaqMan approach on a StepOnePlus instrument (Applied Biosystems, USA). Reactions were loaded onto a 96-well plate for each gene tested. Each experiment was carried out in duplicate, with each well containing the primers for the amplification of the endogenous control DNA (45S) and for the gene of interest. The amplification for each test sample, non-template control, or pool sample was performed in triplicate. Primers for RT-PCR were obtained from Applied Biosystems. All data were normalized to 18S rRNA and quantitative measures obtained using the Δ-Δ-CT method.

### Statistical analysis

Univariate analysis was performed within Prism 6.02 (Graphpad) and Excel (Microsoft, USA). Values were compared using the Student’s t-test or ANOVA as appropriate. For multiple comparisons the Bonferoni correction was used and the significance level stated for these comparisons (p_i_ ≤ α/m, where α is the desired familywise error, m is the number of tests performed and p_i_ is the corrected *p* value for individual tests). In addition, multivariate statistics were used to visualise the lipidomic changes simultaneously using latent variables for the visualisation, including the multivariate approaches of principal components analysis (PCA), partial least squares discriminate analysis (PLS-DA) and orthogonal two partial least squares discriminate analysis (O2PLS-DA) [13, 14] within the SIMCA-P 13.0 software suite (Umetrics, Umea, Sweden). For ease of interpretation, O2PLS-DA plots are reported in the manuscript as these maximise separation between two groups in the first component, with the second component being generated representing variation that is not associated with class membership. Variables associated with this separation were identified using the variable importance parameters for the plot and the associated S-plots [13]. Model validity was assessed by consulting the associated goodness of fit of the model (Q^2^), along with the measures of CV-ANOVA and sample prediction from within SIMCA (Umetrics, Umea, Sweden) [15].

## Results

### Characteristics of the participants

Fasting plasma samples were collected from participants at the same time following a standardised evening meal on the prior evening. The healthy control group was age- and body mass index-matched to both the seven patients with loss-of-function mutations in the *INSR* gene (Table 1) and 14 patients with congenital LD (Table 1). Twelve of the patients with congenital LD had familial partial LD due to either LMNA (lamin A/C; n = 9) or PPARG (peroxisome proliferator activated receptor gamma; n = 3) mutations (Table 1). The other two had congenital generalised LD. All patients had acanthosis nigricans, a cutaneous marker of severe IR, and the typical biochemical profile of patients with either primary INSR defects (severe hyperinsulinaemia, normal TAG and HDL-cholesterol levels, usually with preserved or elevated serum adiponectin levels) or typical biochemical profiles of patients with congenital LD (severe hyperinsulinaemia, hypertriglyceridaemia, low HDL-cholesterol, hypoadiponectinaemia and hypoleptinaemia) (Table 1).

### Comparison of the intact lipid fingerprint

**Fig. 1:**
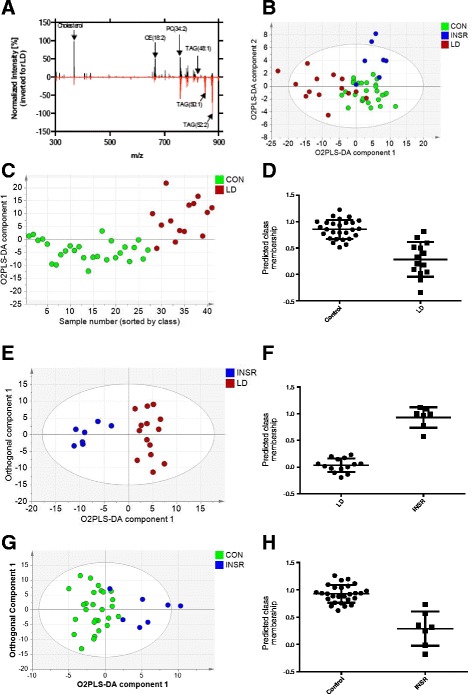
Analysis of intact lipids in the blood plasma of individuals with lipodystrophy or insulin receptoropathies compared with matched controls. **a** Two typical MS spectra from a control (in *black*) and an individual with lipodystrophy (in *red*, shown inverted). **b** O2PLS-DA analysis of the intact lipid dataset for the control (*CON*), lipodystrophic (*LD*) and insulin receptoropathy (*INSR*) groups (48 individuals in total; controls = 27, LD = 14, INSR = 7). The first two O2PLS-DA components are shown (R^2^(X) = 49.5%; R^2^(Y) = 51.5%; Q^2^ = 35.8%; two O2PLS-DA components were fitted). **c** O2PLS-DA model of the control and LD groups. Note only one component is shown as this was the only component that passed validation (the x-axis is arbitrary and the order applied is purely for ease of viewing; R^2^(X) = 45.6%; R^2^(Y) = 57.4%; Q^2^ = 52.6%; one O2PLS-DA component fitted). **d** Predicted class membership of the two groups in (**c**). Control samples should score 1 and LD samples should score 0 in a perfect model. **e** O2PLS-DA model of the INSR and LD groups. One O2PLS-DA component is shown alongside an orthogonal component (R^2^(X) = 74.7%; R^2^(Y) = 89.7%; Q^2^ = 62.8%; one O2PLS-DA component and three orthogonal components fitted). **f** Predicted class membership for the model in (**e**). LD samples should score 1 and INSR samples should score 0 in a perfect model. **g** O2PLS-DA model of the control and INSR groups. One O2PLS-DA component and one orthogonal component is shown (R^2^(X) = 36.4%; R^2^(Y) = 63.8%; Q^2^ = 33.2%; one O2PLS-DA component and one orthogonal component fitted). **h** Predicted class membership for the model in (**g**). Control samples should score 1 and INSR samples should score 0 in a perfect model

We have previously described the strikingly different lipoprotein profiles of patients with LD and INSR, with the latter group being remarkably normal despite severe IR [7]. As expected, VLDL levels were significantly increased and HDL-cholesterol concentrations significantly reduced in the LD group (Table 1). All samples were subjected to liquid/liquid extraction and the organic phase was infused directly into a benchtop orbitrap mass spectrometer, using chip-based nanospray. Figure 1a depicts the mass region between 300 and 900 m/z of two exemplary plasma lipid profiles, acquired for one control and one LD individual, and highlights what was detected in the overall group analyses, with lower concentrations of sterol lipids and cholesterol esters but significantly increased TAG concentrations in the LD group. In total, in positive ion mode 25 diacylglycerides (LipidMaps classification LMGL02), 40 triglycerides (LMGL03), 55 phosphocholines (LMGP01), 51 phosphoserines (LMGP03), 9 phosphoinositols (LMGP06), 26 sphingomyelins (LMSP), and 30 sterols (LMST) were potentially detected according to a database search of the identified peaks across the dataset. We used multivariate statistical methods to investigate the relative changes in the lipid fingerprint across all patients in more detail. O2PLS-DA analysis was unable to separate all three patient groups simultaneously, although the LD group showed clear differences from the other two groups (R^2^(X) = 49.5%; R^2^(Y) = 51.5%; Q^2^ = 35.8% for the model; Fig. 1b). Two-way comparison confirmed the significant differences between the controls and the LD group (R^2^(X) = 45.6%; R^2^(Y) = 57.4%; Q^2^ = 52.6%; Fig. 1c). This model passed cross-validation by CV-ANOVA (*p* = 0.0000007) with the two groups readily separated in terms of predicted Y value (Fig. 1d). The five most discriminating ions in this global assessment were free cholesterol (m/z = 369.351: C_27_H_46_-H_2_O+H^+^; higher in controls and derived from free cholesterol and fragmented cholesterol esters), TAG(52:2) (m/z = 876.802: C_55_H_102_O_6_+NH_4_^+^; increased in LD), CE(18:2) (m/z = 666.618: C_45_H_76_O_2_+NH_4_^+^; increased in controls), TAG(52:3) (m/z = 874.786: C_55_H_100_O_6_+NH_4_^+^; increased in LD) and TAG(50:1) (m/z = 850.786: C_53_H_100_O_6_+NH_4_^+^; increased in LD). A similar discriminatory model with higher statistical significance was produced between the INSR and the LD group (R^2^(X) = 74.7%; R^2^(Y) = 89.7%; Q^2^ = 62.8%) (Fig. 1e). While the model did not pass cross-validation by CV-ANOVA (*p* = 0.07), the two groups were readily separated in terms of predicted Y value (control score = 0.83 ± 0.21; LD score = 0.31 ± 0.30; *p* = 7.9 × 10^−8^ for a significant difference between the two scores) (Fig. 1f). The comparison between the IR and the control group produced the weakest two-group comparison (Fig. 1g; R^2^(X) = 36.4%; R^2^(Y) = 63.8%; Q^2^ = 33.2%), but the model passed cross-validation by CV-ANOVA (*p* = 0.02), and the two groups were partially separated by predicted Y values (control score = 0.92 ± 0.17; LD score = 0.32 ± 0.32; *p* = 1.0 × 10^−7^; Fig. 1h). The five most discriminating ions in this global assessment were sphingomyelin SM(36:1) (m/z = 731.606: C_41_H_83_N_2_O_6_P+H^+^; increased in INSR), SM(18:1/18:1) (m/z = 729.591: C_41_H_81_N_2_O_6_P+H^+^; increased in INSR), PC(36:4) (m/z = 782.569: C_44_H_80_NO_8_P+H^+^; increased in INSR), lyso-phosphocholine (increased in control) and an unidentified peak (exact mass 827.699; increased in INSR). Similarly, the LD and INSR groups could be readily separated by O2PLS-DA (Figure S1 in Additional file 1).

### Investigation of TAG pool changes

To further investigate the observed changes in TAGs we performed a targeted analysis of relative changes within the TAG pool. A total of 40 different TAG species were detected in protonated, sodiated and ammoniated ionisation form and ranged from a total of 46 to 58 carbons in the acyl chains and from a fully saturated state to a maximum of ten double bonds. In order to reveal relative changes in TAG pool composition we normalised the individual TAG profile against the total TAG signal, resulting in relative proportions for each TAG ionisation product.

**Fig. 2:**
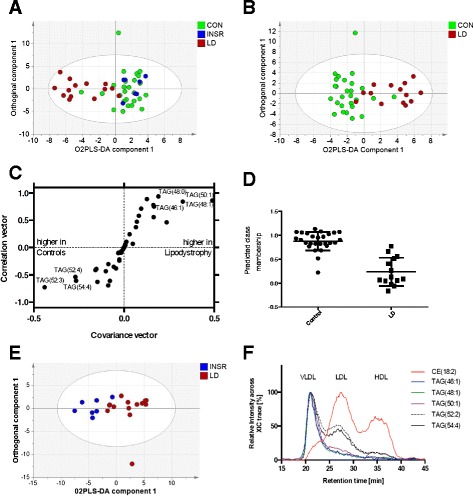
Analysis of the TAG component of the lipid profile from blood plasma of individuals with lipodystrophy or insulin receptoropathies compared with matched controls. **a** O2PLS-DA analysis of the triglyceride dataset for the control (*CON*), lipodystrophic (*LD*) and insulin receptoropathy (*INSR*) groups (48 individuals in total; controls = 27, LD = 14, INSR = 7). One O2PLS-DA component and one orthogonal component are shown (R^2^(X) = 86.6%; R^2^(Y) = 33.5%; Q^2^ = 26.3%; one O2PLS-DA component and five orthogonal components fitted). **b** O2PLS-DA analysis of the triglyceride dataset for the control and LD groups. One O2PLS-DA component and one orthogonal component are shown. (R^2^(X) = 77.8%; R^2^(Y) = 64.1%; Q^2^ = 51.7%; one O2PLS-DA component and three orthogonal components fitted) **c** S-Plot of the O2-PLSDA analysis in (**b**) showing the most discriminatory TAG species for the separation. **d** Predicted class membership for the model in (**b**). In a perfect model control samples would score 1 and the LD samples score 0. **e** O2PLS-DA analysis of the triglyceride dataset for the LD and INSR groups. One O2PLS-DA component and one orthogonal component are shown. (R^2^(X) = 61.4%; R^2^(Y) = 63.4%; Q^2^ = 57.0%; one O2PLS-DA component and one orthogonal component fitted). **f** The relative distribution of triglycerides across the different lipoprotein fractions in human plasma, based on size exclusion chromatography coupled to high resolution mass spectrometry using in-line automated liquid-liquid extraction. The cholersteryl ester CE(18:2) (666.618 m/z ± 5 ppm) shows the distribution of the different lipoprotein fractions. Smaller saturated triglycerides [TAG(46:1) (794.721 m/z ± 5 ppm), TAG(48:1) (822.752 m/z ± 5 ppm) and TAG(50:1) (850.785 m/z ± 5 ppm)] are specific for the VLDL fraction coming from the liver, while larger triglycerides [TAG(52:2) (876.800 m/z ± 5 ppm) and TAG(54:4) (900.800 m/z ± 5 ppm)] can also be found in the LDL fraction and therefore not only originating from the liver

O2PLS-DA analysis on all three phenotypes simultaneously failed to reliably separate them but once again the LD group clustered separately from the other two groups (R^2^(X) = 86.6%; R^2^(Y) = 33.5%; Q^2^ = 26.3%; Fig. 2a). Subsequent two-way comparison demonstrated separation of the LD and control groups (R^2^(X) = 77.8%; R^2^(Y) = 64.1%; Q^2^ = 51.7%; Fig. 2b). Inspection of the corresponding S-plots (Fig. 2c) revealed that TAG species including TAG(50:1), TAG(48:1), TAG(48:0) and TAG(46:1) were increased in LD patients whereas longer, more unsaturated TAGs (including TAG(52:3), TAG(54:4) and TAG(52:4)) were enriched in the control group. The model passed cross-validation according to CV-ANOVA (*p* = 0.001) and the two groups were separated according to predicted Y (control score = 0.87 ± 0.19; LD score = 0.23 ± 0.29; *p* = 3.4 × 10^−10^; Fig. 2d). No model could be built that discriminated the INSR group from controls according to the TAG profiles (data not shown). However, the LD and INSR groups were readily separated by O2PLS-DA (R^2^(X) = 61.4%; R^2^(Y) = 63.4%; Q^2^ = 57.0%; Fig. 2e). The model passed validation according to CV-ANOVA (*p* = 0.006) and the two groups were readily separated according to predicted Y (data not shown). The separation was associated with increases in TAG(48:1), TAG(46:1), TAG(48:0), TAG(50:1) and TAG(46:0) in the LD group and increases in TAG(52:3), TAG(54:4), TAG(54:5), TAG(52:4) and TAG(53:3) in the INSR group as determined by the S-plot associated with the O2PLS-DA plot (data not shown).

**Fig. 3:**
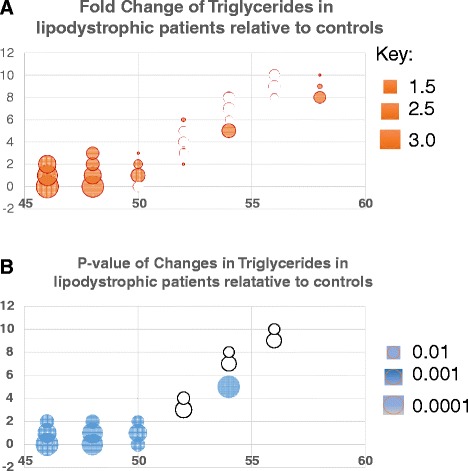
Summary of triglyceride (TAG) changes for even numbers of carbons between the lipodystrophy (LD) and control groups. **a** Bubble plot of fold changes for TAGs. Filled bubbles indicate an increase in concentration in the LD group, open bubbles a decrease in concentration. The area of each bubble is proportional to the log_2_(fold change). **b** Bubble plot of *p* values for significance of change in a given TAG species between the two groups (Student’s t-test). The area of the bubble is proportional to log_10_ of the *p* value. Filled bubbles indicate a significant increase, open bubbles a significant decrease. Note only significant changes are shown as bubbles

To further investigate these TAG changes, univariate analysis was performed on the dataset, comparing both LD against controls and INSR against controls. While only one triglyceride was significantly different in the INSR and control group comparison (TAG(56:8) *p* = 0.0497, 16% increase in INSR but did not pass a Bonferoni correction), 17 TAG species were significantly different between the LD and control groups. This was characterised by an increase in TAGs containing relatively shorter but saturated fatty acids in the LD group, along with a decrease in a number of TAGs containing polyunsaturated fatty acids (Fig. 3; Table S1 in Additional file 1).

The LD group was dominated by females, in keeping with the ease in diagnosing the disorder in females compared with males because of differences in body shape. To ensure the changes in triglyceride profiles did not arise from differences in the gender composition of the control, LD and INSR groups we performed the same analysis on just the females in the dataset. Applying O2PLS-DA to the three groups, the LD group was found to be separate from the two other groups, although the model parameters were relatively poor (R^2^(X) = 36.1%; R^2^(Y) = 25.3%; Q^2^ = 19.5%; data not shown). However, building a model for the LD and control groups readily discriminated the two, with this separation again caused by increases in the concentrations of TAG(48:0), TAG(46:0), TAG(48:1), and TAG(50:1) and relative decreases in TAG(52:3), TAG(53:3), TAG(51:3), TAG(54:7) and TAG (54:6) for the LD group (R^2^(X) = 50.8%; R^2^(Y) = 61.7%, Q^2^ =30.5%; Figure S2 in Additional file 1).

To elucidate the fatty acid composition of the significantly increased TAG species in LD patients we performed fragmentation coupled to chromatographic analysis of the lipid fraction using LC-MS/MS. This demonstrated that the TAGs responsible for the discrimination of the LD group contained palmitic and, to a lesser extent, myristic acid (Table S1 in Additional file 1). In keeping with these observations we also observed that TAGs containing relatively short chain, saturated fatty acids [TAG(46:1), TAG(48:1) and TAG(50:1)] were highly enriched in the VLDL fraction, while TAGs with essential fatty acids were also present in the LDL fraction. We demonstrated this with the help of size exclusion chromatography [16] coupled to high resolution MS using in-line automated liquid-liquid extraction (Fig. 2f). TAGs enriched in palmitic acid and myristic acid are consistent with increased DNL of fatty acids from carbohydrates (for a review of this subject please see [17]).

**Fig. 4:**
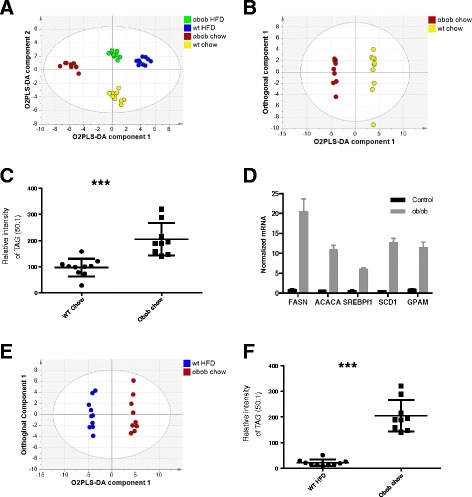
Triglyceride profiles of mouse models associated with de novo lipogenesis. **a** O2PLS-DA analysis of the triglyceride profile of blood plasma from 4-month-old wild-type (*wt*) and ob/ob mice on either a chow or high fat diet (*HFD*). The first two OPLS-DA components are shown (n = 9 per group; R^2^(X) = 71.6%; R^2^(Y) = 94.8%; Q^2^ = 89.2%; three O2PLS-DA components and two orthogonal components were fitted). **b** O2PLS-DA analysis of the triglyceride profile of blood plasma from wild-type and ob/ob mice on a chow diet. The first O2PLS-DA component and orthogonal component are shown (R^2^(X) = 71.8%; R^2^(Y) = 99.8%; Q^2^ = 96.4%; one O2PLS-DA component and three orthogonal components were fitted). **c** Relative intensity of blood plasma TAG(50:1) in 4-month-old wild-type and ob/ob mice under high fat diet (*HFD*) and chow diet (n = 9 for each group; 2.1-fold in the ob/ob mice compared with the wild type on a chow diet (*p* = 0.0002)). **d** Increased expression of genes involved in DNL in the liver of ob/ob mice compared with wild-type controls as measured by RT-PCR. Expression of DNL genes in liver from 4-month-old wild-type control mice (n = 6) and ob/ob mice (n = 6). Data are represented as mean ± standard error of the mean and all data were normalized to 18S rRNA. Two-way ANOVA, *p* < 0.0001. *FASN* fatty acid synthase, *ACACA* acetyl-CoA carboxylase 1, *SREBP1F* sterol regulatory element binding protein-1c, *SCD1* stearoyl-coenzyme A desaturase 1, *GPAM* glycerol-3-phosphate acyltransferase, mitochondrial. **e** O2PLS-DA analysis of the triglyceride profile of blood plasma from wild-type mice on a high fat diet (*HFD*; low de novo lipogenesis) and ob/ob mice on a chow diet (high de novo lipogenesis). **f** Relative intensity of blood plasma TAG(50:1) in 4-month-old wild-type mice on a high fat diet and ob/ob mice on a chow diet (n = 9 for each group). ***p < 0.001 according to Student’s t-test

To further explore the impact DNL has on the TAG profile, we examined blood plasma TAGs in 4-month-old wild-type and ob/ob mice fed a high carbohydrate (chow) or high fat diet. It has previously been shown that DNL is very significantly increased in chow-fed ob/ob mice [18, 19] and reduced in mice fed high fat diets. DIMS detected 45 triglycerides, with both the total lipid profile and the TAG complement readily discriminating the mice according to genotype and diet (R^2^(X) = 71.6%; R^2^(Y) = 94.8%; Q^2^ = 89.2%; Fig. 4a). Comparing the two chow diet groups, ob/ob mice were readily separated from control animals using O2PLS-DA (R^2^(X) = 71.8%; R^2^(Y) = 99.8%; Q^2^ = 96.4%; Fig. 4b). We observed that TAG(50:1) was increased 2.1-fold in the ob/ob mice compared with the wild type on a chow diet (*p* = 0.0002; Fig. 4c), and, in general, TAGs containing shorter chain and more saturated fatty acids were increased in the ob/ob mice fed a carbohydrate diet (TAG(52:2), TAG(54:3), TAG(56:3), TAG(50:1) and TAG(52:2) were increased in ob/ob mice and TAG(52:5), TAG(52:4), TAG(54:7), TAG(52:6) and TAG(56:8) were increased in the wild-type mice). To confirm that these TAG changes were associated with increased DNL, the concentration of transcripts of key enzymes in DNL were measured by RT-PCR (Fig. 4d). The expression of fatty acid synthase, acetyl-CoA carboxylase 1, sterol regulatory element binding protein-1c, stearoyl-CoA desaturase 1 and glycerol-3-phosphate acyltransferase were all significantly increased (two-way ANOVA, *p* < 0.0001).

Given that a high fat diet suppresses DNL in the liver, we next compared wild type mice on a high fat diet with ob/ob mice on a chow diet where DNL is expected to be most raised. O2PLS-DA of the TAG profile produced a robust model that readily discriminated the two groups (R^2^(X) = 63.9%; R^2^(Y) = 99.3%; Q^2^ = 98.1%; Fig. 4e), and the model readily passed cross-validation using CV-ANOVA (*p* = 7 × 10^−12^). The ob/ob chow diet group was associated with increases in TAG(52:2), TAG(54:3), TAG(50:2), TAG(50:1) and TAG(56:3), while the wild-type chow diet group was associated with increases in TAG(52:4), TAG(54:6), TAG(54:5), TAG(52:4) and TAG(54:7). This separation was associated with a 4.2-fold increase in TAG(50:1) in the ob/ob chow diet group compared with the wild-type high fat diet group (Fig. 4f).

## Discussion

The relative enrichment in shorter chain saturated TAG species observed in samples from the LD group is remarkably similar to that recently shown to be associated with IR in a large epidemiological study [1] and smaller selected cohort studies [2, 20]. This pattern was also observed in samples from five healthy men fed a hypercaloric carbohydrate-enriched diet specifically designed to stimulate hepatic DNL [21]. These data are also consistent with stable isotope-based measurements of the rate of DNL in patients with LD and *INSR* mutations [7]. In this previous study DNL was found to increase over threefold in lipodystrophic individuals compared with the control group, with those with insulin receptoropathies and mutations in AKT2 being intermediate in terms of their DNL capacity. Although detailed physiological measurements of the relative contributions of adipose tissue lipids, dietary lipids and DNL-derived lipids to liver and plasma TAGs suggest that lipolytically derived fatty acids constitute by far the greatest source of lipids [22], our data imply that increased DNL significantly changes the composition of the triglycerides in lipodystrophic patients and probably also in other prevalent forms of the metabolic syndrome. Furthermore, our assay used to measure triglycerides associated with DNL in the present study took only 3 min to perform and required only 15 μl of blood plasma compared with a two day intervention study to measure DNL using stable isotopes. This allowed us to measure groups of much larger size than in [7], and also demonstrate that increased DNL was something specific to the LD group and not found in the INSR group in our larger powered study. This suggests that raised DNL is associated with increased glucose uptake into the liver as a result of a failure of adipose tissue uptake in LD patients, rather than a mechanism that is associated with the downstream effects of IR (and would be common to both LD and INSR patients).

To further investigate DNL we conducted an animal study to directly manipulate DNL and investigate the liver directly. We examined the effect of a chow diet, relatively high in carbohydrates, on DNL compared with a high fat diet that suppresses DNL in both wild-type and ob/ob mice. The ob/ob mouse with its increased appetite because of a lack of leptin would be expected to provide a more extreme phenotype on both diets compared with its wild-type control. We were able to demonstrate transcriptionally that DNL is markedly increased in ob/ob mice compared with wild type on a chow diet as has been previously reported [23], and this was associated with a relative increase in TAGs containing shorter, more saturated fatty acids as in the LD group. However, the lipidomic changes were not directly recapitulated as in the mouse study a more limited number of TAGs were detected compared with humans. In our previous studies we have found that mice have a more limited set of TAGs typically within their blood plasma [24–27], which may reflect the more limited diet mice receive as well as genetic differences between the species. One of the most discriminatory lipids was TAG(50:1), with a fourfold difference in this lipid species between the wild-type mice on a high fat diet, which would be expected to have the most suppressed DNL capability, and ob/ob mice on a chow diet, which has the highest capacity for DNL in this mouse study. Furthermore, although obese, ob/ob mice still have functional adipose tissue and consumed carbohydrate will be taken up by both adipose tissue and the liver, reducing the impact of DNL on the blood plasma lipidome.

Dyslipidaemia is a common feature of a number of metabolic diseases and it is usual for total triglyceride concentrations to be measured as part of patient assessment. However, these results demonstrate that not all triglycerides should be considered together and certain species may act as proxies for specific biological processes, in particular those containing shorter, more saturated fatty acids which may reflect DNL. The selective increase in TAGs that contain shorter, more saturated fatty acids has been found in a number of cohort studies examining both T2DM and CVD [1–3]. These studies, together with our current study, strongly suggest that DNL may make a significant contribution to the relative risk of developing metabolic diseases. Furthermore, the measurement of individual TAG species may be particularly appropriate to the application of precision medicine for sufferers with aspects of the metabolic syndrome. For example, while glucokinase agonists have been shown to reduce hyperglycaemia dramatically in type 2 diabetics, they are also associated with NAFLD in a subset of patients. However, Dhanesha and colleagues have demonstrated that NAFLD can be reduced in db/db mice if a glucokinase agonist is co-administered with exendin-4, a GLP-1 agonist [28]. Thus, a lipidomic assay for flux through DNL could be used to assess the use of glucokinase agonists either with or without a GLP-1 agonist.

Recently, together with Forouhi and colleagues [29], we examined the fatty acid content of phospholipids in blood plasma by gas chromatography to examine the association of specific fatty acids with relative risk of developing T2DM across over 28,000 individuals in EPIC-InterAct. We found that myristic, palmitic and staeric acid were all associated with increased risk of developing T2DM. In addition, we examined which foods were associated with these fatty acids as reported in food frequency questionnaires, with alcohol, potato consumption, margarine and sugary drinks being most associated with these saturated fatty acids. With the exception of margarine consumption, the other three food substances are all associated with DNL, suggesting that foods that promote DNL may be responsible for increased risk of T2DM. This emphasizes the importance of considering individual lipid species rather than considering either all saturated fatty acids or all triglyceride species together. It also raises concerns in terms of the development of fatty liver disease in the general population given the cumulative health advice over the last ~40 years to reduce saturated fatty acid intake, which in turn has led to an increase in sugar intake in many diets.

## Conclusions

Plasma lipid profiling in patients with LD and *INSR* mutations indicates that the relative enrichment of shorter chain saturated TAG species associated with human IR in previous studies is not purely a secondary consequence of defective insulin action. Our observations related to changes in the lipid profile of patients with LD are also consistent with the notion that this lipidomic pattern is a useful marker of IR likely to reflect increased hepatic DNL and that it can develop as a consequence of adipose tissue dysfunction. The increase in DNL is most likely to be occurring in the liver given the reduced adiposity in LD, although our own data do not specifically address the site of DNL. These changes can be recapitulated in mouse models of DNL, with the caveat that humans appear to produce a wider range of TAGs in terms of both fatty acid chain length and degree of unsaturation.

## Additional file

Additional file 1:
**Supplementary Table S1 and Figures S1 and S2.** (DOCX 102 kb)

## Abbreviations

CVD: cardiovascular disease
DNL: de novo lipogenesis
HDL: high-density lipoprotein
INSR: insulin receptoropathies
IR: insulin resistance
LC: liquid crystallography
LD: lipodystrophy
MS: mass spectrometry
NAFLD: non-alcoholic fatty liver disease
O2PLS-DA: orthogonal two partial least squares discriminate analysis
PBS: phosphate-buffered saline
QC: quality control
T2DM: type 2 diabetes mellitus
TAG: triacylglycerol
VLDL: very-low-density lipoprotein
