# Supplementary material for: Mechanistic insights revealed by lipid profiling in monogenic insulin resistance syndromes
Source: Genome Med. 2015 Jun 28;7(1):63. doi: 10.1186/s13073-015-0179-6 (PMC4535665; doi:10.1186/s13073-015-0179-6)
Supplement: Additional file 1: — Supplementary Table S1 and Figures S1 and S2. (DOCX 102 kb) [file 13073_2015_179_MOESM1_ESM.docx]

**Supplementary data: Mechanistic insights revealed by lipid profiling in monogenic insulin resistance syndromes.**

Michael Eiden^1^, Albert Koulman^1^, Mensud Hatunic^2^, James A. West^1,3^, Steven Murfitt^3^, Michael Osei^1^, Claire Adams^2^, Xinzhu Wang^1,3^, Yajing Chu^3^, Luke Marney^1^, Lee D. Roberts^1,3^, Stephen O’Rahilly^2^, Robert K. Semple^2^, David B. Savage^2^, and Julian L. Griffin^1,3^

*^1^Medical Research Council Human Nutrition Research, Fulbourn Road, Cambridge;^2^Metabolic Research Laboratories, Institute of Metabolic Science, University of Cambridge, UK; ^3^Department of Biochemistry and the Cambridge Systems Biology Centre, University of Cambridge, Tennis Court Road, Cambridge.*

**Supplementary Table 1:** Triglyceride (TAG) species in blood plasma from individuals with lipodystrophy (LD) or the control group. Each TAG species is identified by the number of carbons (first) and double bonds (second). Fold-change indicates a relative increase in the LD group. P-value calculated for a Student’s T-test comparing the two groups. Bonferoni corrected p_i_ = 0.0018

| **TAG species** | **m/z** | **Rt** | **HRMS^2^ fragmentation** | **MSI identification level ^[^**[**^25^**](#_ENREF_25)**^]^** | **Fold-change** | **p-value** |
| --- | --- | --- | --- | --- | --- | --- |
| **46:0 (M+NH_4_^+^)** | 796.739 | 42.1 | 523.473(100); 525.477(2); 551.504(47); 579.53(5) | 2 | 3.13 | 0.0000001 |
| **46:1 (M+NH_4_^+^)** | 794.723 | 40.4 | 493.426 (24); 495.440(12); 519.441(24); 521.456(100); 533.456(6); 535.472(21); 549.488(38) | 2 | 2.55 | 0.00001 |
| **46:2 (M+NH_4_^+^)** | 792.707 | 38.6 | 493.426 (24); 495.440(12); 519.441(24); 521.456(100); 533.456(6); 535.472(21); 549.488(38) | 2 | 2.04 | 0.0019 |
| **48:0 (M+NH_4_^+^)** | 824.771 | 43.6 | 523.471(9); 537.489(5); 551.502(100), 565.519(5); 579.535(7) | 2 | 2.89 | 0.0000008 |
| **48:1 (M+NH_4_^+^)** | 822.758 | 42.1 | 523.471(42), 549.487(100), 551,502(29), 577.519(27) | 2 | 1.96 | 0.000002 |
| **48:2 (M+NH_4_^+^)** | 820.739 | 40.4 | 521.456(20); 547.472(62); 549.487(100); 575.503(5); 577.516(7) | 2 | 1.47 | 0.002 |
| **48:3 (M+NH_4_^+^)** | 818.724 | 38.7 | 519.441(43); 521.457(100); 523.473(21); 545.457(43); 547.473(61); 549.488(8); 573.488(54); 575.504(28); 577.519(5); 601.52(40); 603.531(2) | 2 | 1.42 | 0.036 |
| **50:0 (M+NH_4_^+^)** | 852.802 | 45.0 | 495.443(1); 523.473(3); 537.488(2); 551.503(61); 565.519(15); 579.535(100) | 2 | 0.8 | 0.002 |
| **50:1 (M+NH_4_^+^)** | 850.786 | 43.6 | 549.486(4); 551.501(66); 552.504(11); 577.517(100); 579.532(2); 605.548(2) | 2 | 1.57 | 0.00003 |
| **50:2 (M+NH_4_^+^)** | 848.769 | 42.0 | 547.471(1); 549.485(100); 551.501(8); 563.501(0); 575.501(65); 577.516(42); 579.53(0); 603.532(25) | 2 | 1.19 | 0.009 |
| **50:3 (M+NH_4_^+^)** | 846.754 | 40.4 | 547.47(77); 549.485(100); 549.56(0); 549.578(0); 561.486(0); 563.504(0); 573.485(60); 575.501(81); 577.516(2); 601.516(62); 603.532(4) | 2 | 1.014 | 0.85 |
| **50:4 (M+NH_4_^+^)** | 844.739 | 39.1 | 545.455(15); 546.034(0); 547.47(100); 549.485(18); 571.47(7); 573.486(34); 575.502(3); 597.271(0); 599.501(75); 601.517(6) | 2 | 1 | 0.97 |
| **52:2 (M+NH_4_^+^)** | 876.801 | 43.6 | 575.502(9); 577.517(100); 579.532(10); 603.532(64); 605.548(2) | 2 | 1.013 | 0.786 |
| **52:3 (M+NH_4_^+^)** | 874.785 | 42.3 | 549.485(1); 573.486(1); 575.502(100); 577.517(72); 601.518(88); 603.533(20) | 2 | 0.85 | 0.000281 |
| **52:4 (M+NH_4_^+^)** | 872.769 | 40.9 | 573.486(35); 575.502(100); 577.517(6); 599.502(53); 601.518(34); 603.533(1) | 2 | 0.81 | 0.00989 |
| **52:5 (M+NH_4_^+^)** | 870.754 | 39.5 | 547.472(2); 549.489(20); 551.503(6); 571.473(28); 573.488(100); 575.504(86); 577.52(9); 597.489(65); 599.504(58); 601.52(7); 603.535(3) | 2 | 0.83 | 0.15 |
| **52:6 (M+NH_4_^+^)** | 868.739 | 38.4 | 523.473(49); 547.471(22); 549.489(31); 571.473(54); 573.489(100); 575.505(19); 595.472(30); 597.489(60); 599.503(13); 603.534(10);625.516(7); 627.529(5) | 2 | 1.04 | 0.76 |
| **54:5 (M+NH_4_^+^)** | 898.785 | 40.9 | 549.488(2); 551.506(4); 553.511(1); 573.489(1); 575.505(12); 577.52(39); 579.53(1); 597.49(4); 599.505(100); 601.52(100); 603.535(30); 625.52(20); 627.536(3) | 2 | 1.56 | 0.03 |
| **54:6 (M+NH_4_^+^)** | 896.770 | 39.5 | 549.49(9); 551.504(33); 573.489(4); 575.504(65); 577.52(23); 595.473(1); 597.488(48); 599.504(100); 601.52(47); 603.536(5); 623.504(29); 625.519(8) | 2 | 0.88 | 0.11 |
| **54:7 (M+NH_4_^+^)** | 894.754 | 38.0 | 549.489(100);573.489(36); 575.504(48); 595.474(13); 597.489(60); 599.504(49); 601.518(8); 621.489(17); 623.504(15); 651.528(2); 653.604(8) | 2 | 0.76 | 0.0013 |
| **54:8 (M+NH_4_^+^)** |  |  |  | 3 | 0.78 | 0.03 |
| **56:8 (M+NH_4_^+^)** | 920.770 | 39.6 | 575.504(100); 577.52(1); 599.504(14); 601.519(11); 621.489(5); 623.504(18); 625.518(3); 647.504(14); 649.519(3) | 2 | 0.88 | 0.11 |
| **56:9 (M+NH_4_^+^)** | 918.754 | 33.7 | 573.487(96); 597.487(100); 599.503(83) | 2 | 0.76 | 0.0013 |
| **56:10 (M+NH_4_^+^)** | 916.739 |  |  | 3 | 0.78 | 0.03 |
| **58:8 (M+NH_4_^+^)** | 948.801 | 41.0 | 575.503(6); 601.519(15); 602.522(5); 603.536(100); 627.534(8); 649.52(25); 651.536(3) | 2 | 1.36 | 0.097 |
| **58:9 (M+NH_4_^+^)** | 946.786 | 38.1 | 601.52(100); 625.518(8); 647.504(3); 649.52(9) | 2 | 1.05 | 0.79 |
| **58:10 (M+NH_4_^+^)** | 944.770 |  |  | 3 | 1.01 | 0.98 |

**Supplementary Figure 1**

OPLS-DA analysis of the lipid profile of blood plasma from individuals with insulin receptoropathies (INSR) and lipodystrophy (LD). Using this model to predict class membership where 1 signifies a perfect classification as LD and 0 as a perfect classification of INSR, the two groups were readily discriminated with INSR = 0.81 ± 0.22 and LD = 0.09 ± 0.22 (p = 1.2 * 10^-6^ according to a Student’s t-test).


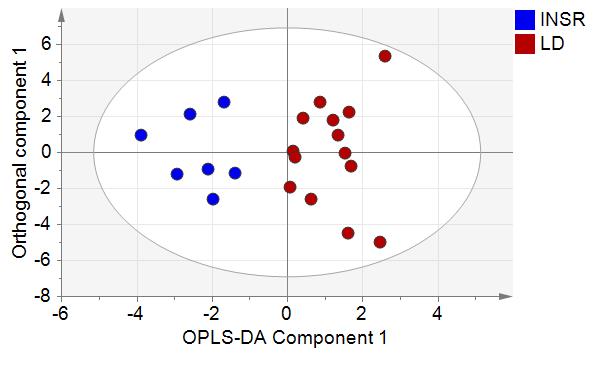


**Supplementary Figure 2**

OPLS-DA analysis of the triglyceride (TAG) profiles of female lipodystrophic patients compared with female controls. A. Scores Plot R^2^X=50.8%; R^2^Y=61.7%, Q^2^=30.5%. B. S-plot showing the most important TAGs for discriminating the two groups.

**A**


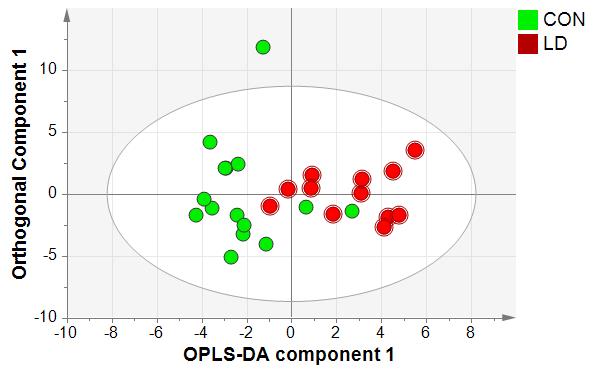


**B
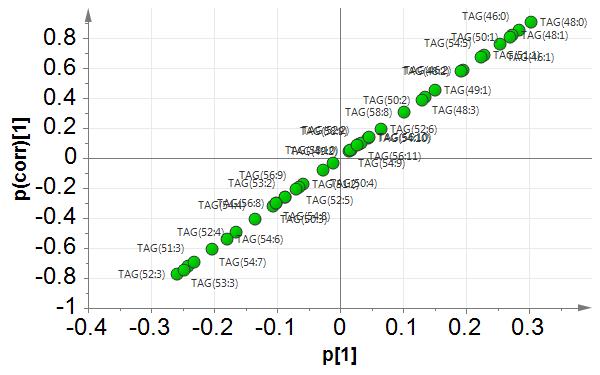
**
